# A Comprehensive Review on the Antibacterial, Antifungal, Antiviral, and Antiparasitic Potential of Silybin

**DOI:** 10.3390/antibiotics13111091

**Published:** 2024-11-15

**Authors:** José Lima Pereira-Filho, Amanda Graziela Gonçalves Mendes, Carmem Duarte Lima Campos, Israel Viegas Moreira, Cinara Regina Aragão Vieira Monteiro, Suzany Hellen da Silva Soczek, Elizabeth Soares Fernandes, Rafael Cardoso Carvalho, Valério Monteiro-Neto

**Affiliations:** 1 Centro de Ciências da Saúde, Universidade Federal do Maranhão—UFMA, São Luís 65080-805, MA, Brazil; jlp.filho@outlook.com (J.L.P.-F.); amandagrazielamendes@gmail.com (A.G.G.M.); carmemdlcampos@gmail.com (C.D.L.C.); israel.moreira@ufma.br (I.V.M.); cinaraaragao@hotmail.com (C.R.A.V.M.); carvalho.rafael@ufma.br (R.C.C.); 2 Instituto de Pesquisa Pelé Pequeno Príncipe, Curitiba 80250-060, PR, Brazil; suzanyhellen@gmail.com (S.H.d.S.S.); elizabeth.fernandes@pelepequenoprincipe.org.br (E.S.F.); 3 Faculdades Pequeno Príncipe, Curitiba 80250-060, PR, Brazil

**Keywords:** silybin, antibacterial, antifungal, antiviral, antiparasitic, antimicrobial resistance

## Abstract

Silybin, a flavonolignan extracted from the seeds of the plant species *Silybum marianum* (L.) Gaertn., has a variety of pharmacological activities, including antimicrobial activity against several microorganisms of clinical interest. This review analyzes the existing studies on silybin’s antimicrobial activity and possible mechanisms of action. Silybin has been shown to inhibit the growth of Gram-positive and Gram-negative bacteria, as well as some fungi, viruses, and protozoa. In general, possible mechanisms of antimicrobial action include the inhibition of efflux pumps, prevention of biofilm formation, reduction of the expression of virulence factors, induction of apoptosis-like effects, and plasma membrane damage, as well as the inhibition of nucleic acid and protein synthesis. Silybin has been shown to have synergistic effects when combined with conventional antibiotics against both drug-sensitive and drug-resistant microorganisms. However, the low bioavailability observed for this flavonolignan has been a challenge to its clinical use. In this context, nanotechnology has been used to increase silybin’s bioavailability while enhancing its antimicrobial activity. Furthermore, certain structural modifications have been able to enhance its antimicrobial activity in comparison to that of the natural molecule. Overall, this review provides insights into the scientific understanding of the mechanism of action of silybin and its desired properties for the effective treatment of infections.

## 1. Introduction

Antimicrobial resistance is a global threat to human health, and its environmental spread has been documented [1]. The emergence of multidrug-resistant microorganisms has increased the need to develop new strategies to solve the problem of drug resistance [2]. Multidrug resistance (MDR) in microorganisms of clinical interest (bacteria, fungi, viruses, and protozoa) has become a major problem worldwide because of the continued misuse of antimicrobials [3,4,5].

In this context, products of natural origin, including phenolic compounds (e.g., flavonoids) obtained from plants, have received renewed attention due to their diverse chemical structures and bioactive characteristics that may present different mechanisms of action against microbial pathogens, as well as contribute to the reduction of resistance when combined with conventional antibiotics; therefore, they are potential sources of new therapeutic strategies to combat resistant microorganisms [6,7,8].

Flavonoids are phenolic secondary metabolites found in plants and fungi. They usually have beneficial biological effects, such as antioxidant, antimutagenic, and anti-inflammatory activities. Flavonolignans form a small subclass of flavonoids, which are mainly isolated from silymarin (an extract from the seeds of milk thistle *Silybum marianum*) [9].

Silybin, a bioactive phenolic compound present in the extract of the seeds of the plant species *Silybum marianum* (L.) Gaertn., popularly known as milk thistle, has received considerable attention because of its wide range of pharmacological activities, including anti-inflammatory, anticancer, antioxidant, antimicrobial, and hepatoprotective activity [10,11,12,13]. Previous studies have also demonstrated the high potential of silybin to inhibit the growth and viability of several microorganisms, including Gram-negative bacteria [9,14], Gram-positive bacteria [14,15], fungi [11], viruses [16], and protozoa [17]. Thus, its effects as an antimicrobial agent offer new perspectives for the development of alternative therapies against infectious diseases, especially considering the growing emergence of new strains of microorganisms resistant to the traditional antibiotics used in therapy [2].

The increasing number of studies on the antimicrobial effects of silybin calls for a thorough analysis to consolidate the current understanding, emphasize key findings, and pinpoint areas requiring further exploration. Our aim was to scrutinize and contextualize the scientific literature concerning silybin’s antimicrobial capabilities, focusing particularly on understanding its modes of action and assessing its viability as a potential treatment.

To perform this review, a comprehensive search was conducted in several databases, including PubMed, Web of Science, Google Scholar, and Scopus, to identify original studies that provided information on the antimicrobial activities of silybin against bacteria, fungi, viruses, and protozoa. The review of the literature spanned from 1968 to 2024, offering a thorough examination of both historical and contemporary perspectives. To identify relevant publications, specific search terms were employed: “Antimicrobial activity AND silybin”, “Silybin AND antibacterial”, “Silybin AND antifungal”, “Silybin AND antiviral”, “Silybin AND protozoa”, and “Silybin AND drug resistance”. To enhance the comprehensiveness of the review, references cited in the initially identified publications were also gathered. The review process excluded certain types of content, including editorial letters, non-original research, conference papers lacking sufficient detail, non-English articles, and studies without access to the full text. This methodological approach was designed to concentrate on pertinent, detailed, and peer-reviewed research in this field.

## 2. Chemical Characteristics of Silybin

Silybin, also known as silibinin, flavobin, and silymarin I, is the primary flavonolignan found in the silymarin complex extracted from *S. marianum* [10,18]. The initial description and naming of silybin occurred in a pioneering study by Pelter and Hansel in 1968, employing detailed examination of ^1^H-Nuclear Magnetic Resonance (NMR) (100 MHz, DMSO-*d*_6_) and Mass Spectrometry (MS) data [19]. However, the absolute configuration of silybin, particularly at the C-2 and C-3 positions, was not determined until 1975, when the same researchers employed the degradative approach methodology [20]. The chemical structure of silybin is (2R,3R)-3,5,7-trihydroxy-2-[(2R,3R)-3-(4-hydroxy-3-methoxyphenyl)-2-(hydroxymethyl)-2,3-dihydro-1,4-benzodioxin-6-yl]-2,3-dihydrochromen-4-one, with a molecular weight of 482.441 g/mol and molecular formula of C_25_H_22_O_10_. The compound comprises two main components connected by a 1,4-dioxane ring: one derived from taxifolin, a flavonol-type flavonoid, and the other from a phenylpropanoid unit, specifically coniferyl alcohol [21].

Silybin exhibits high stability in acidic environments, including Brønsted acids, but it is less stable under basic conditions or when exposed to Lewis acids. When heated above 100 °C for extended periods, the structure of the compound breaks down. Although silybin demonstrates good resistance to reduction, it readily oxidizes to 2,3-dehydrosilybin when exposed to O_2_ molecules. In neutral pH aqueous solutions, silybin behaves as a weak acid, with pKa values of 6.63 for the 5-OH group, 7.7–7.95 for the 7-OH group, and 11.0 for the 20-OH group [18].

The molecule contains five hydroxyl groups that are the primary targets for derivatization: 3-OH, 5-OH, 7-OH, 20-OH, and 23-OH. Among these, the 5-OH, 7-OH, and 20-OH groups exhibit phenolic properties. The 7-OH group is more reactive than the 20-OH group because of reduced steric hindrance and the presence of hydrogen bonds. The 5-OH group uniquely forms strong hydrogen bonds with the adjacent oxo group, which is conjugated to an aromatic ring and acts as a free-electron donor. The 23-OH group is susceptible to oxidation and esterification with carboxylic acids. As previously mentioned, the 3-OH group readily oxidizes to a ketone upon exposure to atmospheric O_2_ to form 2,3-dehydrosilybine. Silybin has poor solubility in polar protic solvents, such as MeOH and EtOH, and is insoluble in nonpolar solvents like chloroform and petroleum ether. However, it dissolves well in polar aprotic solvents, such as DMF, acetone, THF, and DMSO [22].

**Figure 1:**
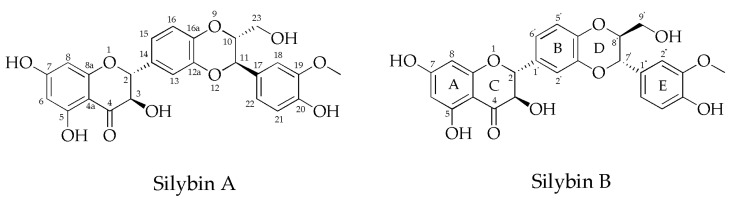
Chemical structure of silybin A and silybin B. Flavonolignans contain a flavonoid moiety linked to a lignan and phenylpropanoid moiety.

Silybin, as found in nature, exists as an equal mixture of two diastereoisomers: silybin A and silybin B, with their chemical structure shown in Figure 1. These isomers have nearly identical ^1^H and ^13^C NMR spectra and lack distinctive signals for individual identification. High-resolution ^13^C NMR spectra of natural silybin reveal two sets of similar signals, and assigning them to specific diastereoisomers is not feasible without authentic standards [23].

High-performance liquid chromatography (HPLC) is the preferred method for separating diastereoisomers. This technique differentiates molecules based on their retention time [21]. HPLC, along with co-chromatography using authentic standards, effectively distinguished silybin A and B [23]. Silybin A, composed of 11R, 10R, 3R, and 2R isomers, has the IUPAC name (2 R,3 R)-2-[(2R,3R)-2,3-dihydro-3-(4-hydroxy-3- methoxyphenyl)-2-(hydroxymethyl)-1,4-benzodioxin-6-yl]-2,3-dihydro-3,5,7-trihydro-4H-1-benzopyran-4-one. Conversely, silybin B, with a configuration of 11S and 10S, 3S, and 2S, and its IUPAC name is (2R,3R)-2-[(2S,3S)-2,3-dihydro3-(4-hydroxy-3-methoxyphenyl)-2-(hydroxymethyl)-1,4-benzodioxin-6-yl]-2,3-dihydro-3,5,7-trihydroxy4H-1-benzopyran-4-one [18]. The diastereoisomers exhibited distinct optical rotations; silybin A showed [α]^23^_D_ + 20.0° (c 0.21, acetone), whereas silybin B displayed [α]^23^_D_ − 1.07° (c 0.28, acetone) [21]. In addition, their crystallization properties differ. Silybin A forms flat yellow crystals when crystallized from MeOH–H_2_O, with a melting point of 162–163 °C. In contrast, silybin B crystallizes as granular yellow crystals in the same solvent and melts at 158–160 °C [18,21].

## 3. Antibacterial Activity

The antibacterial activity of secondary metabolites extracted from plants has been extensively studied over the last few years, as they are a natural source of molecules with varied and complex chemical structures that have a broad spectrum of action against various human pathogens that can serve as prototypes for the development of new antimicrobial agents [24,25].

Silybin is a flavonolignan with antibacterial activity against a wide range of pathogens involved mainly in mucosal, skin, gastrointestinal, and respiratory infections [12,14,26]. Regarding studies on the antibacterial activity of silybin, it was found that this compound has better activity against Gram-positive bacteria, with strains of *S. aureus* [9,14,27,28,29,30,31,32] and methicillin-resistant *S. aureus* (MRSA) being the main representatives [15,26,27,33,34]. The studies describing the antibacterial activity of silybin are summarized in Table 1.

In general, pure compounds are more effective against Gram-positive bacteria than Gram-negative bacteria [45]. This is due to the outer lipopolysaccharide membrane present in the constitution of Gram-negative bacteria. This outer membrane restricts the diffusion of compounds through the covering layer of lipopolysaccharides. In gram-positive bacteria, the compound exerts its effects after direct contact with phospholipids. This effect takes place through the rise in ion diffusion or the leakage of the cell’s vital components [45]. In the study carried out by Lee et al. [35], the antibacterial activity of silybin was verified against eleven oral pathogens with minimum inhibitory concentration (MIC) values ranging from 0.1 to 3.2 μg/mL, including the following: *A. actinomycetemcomitans*, *F. nucleatum*, *P. gingivalis*, *P. intermedia*, *S. anginosus*, *S. criceti*, *S. gordonii*, *S. mutans*, *S. ratti*, *S. sanguinis*, and *S. sobrinus*. Silybin also exhibits antibacterial activity against some Gram-positive pathogens, such as *S. aureus* [9,14,27,28,29,30,31,32,35], MRSA [15,26,27,33,34,43], Methicillin-sensitive *S. aureus* (MSSA) [15], *B. subtilis* [28,29,41], *S. epidermidis* [41], *E. faecium* [27], *E. faecalis* [28,38], and *C. xerosis* [31]. In addition, silybin has also been shown to exert an inhibitory effect against Gram-negative bacteria, such as *E. coli* [14,27,28,29,31,32,36,37,38,39], *P. aeruginosa* [9,14,27,28,29,31,37,38], *K. pneumoniae* [28,38], *A. baumannii* [28], *S. typhi* [31], *V. campbellii* [9], and *P. mirabilis* [28] with an MIC value ranging from 0.312 to 1.024 µg/mL. Furthermore, silybin exhibits inhibitory activity against the resistant strain of *K. oxytoca*; however, this has an MIC too high to be considered relevant for this pathogen, corresponding to 500 mg/mL [40]. In addition to these pathogens, silybin also inhibits *H. pylori* with an MIC value of 256 µg/mL [12]. Chronic *H. pylori* infections are associated with the development of several diseases of the gastrointestinal tract, such as gastric cancer, gastric ulcers, biliary tract cancer, and mucosa-associated lymphoid tissue lymphoma [12].

Silybin is also effective against *M. tuberculosis*, the main pathogen that causes tuberculosis, with an MIC value between 50 and 400 µM [42]. Although tuberculosis treatment has been available for over 60 years, it requires the use of four antibiotics for a prolonged period of at least six months. Anti-tuberculosis treatment carries a risk of developing side effects (including gastric, neurological, and hematological alterations) and is potentially hepatotoxic [42]. It has been reported that silybin has hepatoprotective activity [13,46]; therefore, this compound has promising therapeutic potential for research to consolidate its action as an anti-tuberculosis agent, since it also guarantees hepatoprotective action.

## 4. Antifungal Activity

Despite several studies on the antibacterial activity of silybin against human pathogenic microorganisms, the antifungal activity of this compound has rarely been reported. Previously published studies indicate that silybin has anti-*C. albicans* activity, with MIC values ranging from 4 to 1.024 µg/mL [14,28,29].

Silybin also inhibited (8 µg/mL) *C. parapsilosis* strains [28]. Similarly, Yun and Lee [47] reported antifungal activity against *C. parapsilosis*. Silybin also had inhibitory activity against other non-*albicans Candida* species (NAC) such as strains of *C. glabrata* [29], *C. krusei* [14], and *C. tropicalis* [14,29,48]. Additionally, in addition to species of the genus *Candida*, this compound has an inhibitory effect against other fungal pathogens, such as *A. flavus*, *M. furfur* [47,48], and *T. beigelii* [47]. The studies describing silybin antifungal activity are summarized in Table 2.

## 5. Antiviral Activity

Silybin was shown to present significant antiviral activity in recently published studies. The results indicate that silybin and its derivatives have a wide range of activity against different types of viruses, such as hepatitis B (HBV) [49], hepatitis C (HCV) [28,50,51,52,53,54,55,56,57,58,59,60,61,62,63,64,65,66,67,68,69,70], human immunodeficiency virus (HIV) [52,60,63,71,72], influenza A virus (IAV) [73], *Chikungunya* virus [74], and human enterovirus 68 (EV68) [75]. Silybin was also found to exert antiviral activity against the severe acute respiratory syndrome coronavirus 2 (SARS-CoV-2) virus [16,76,77,78,79].

## 6. Antiparasitic Activity

The antiparasitic activity of silybin is still under studied compared to its antibacterial, antifungal, and antiviral activities. Studies describing its antiparasitic activities are summarized in Table 3. A promising activity of silybin against some parasites was described, mainly those belonging to the genera *Tryfpumps and porins in uropathogen* [80,81] and *Leishmania* [82,83,84]. Silybin also inhibited the growth of *T. cruzi* epimastigotes (at an IC_50_ of 25 µM) and amastigotes (at an IC_50_ of 79.81 µM). However, silybin monotherapy was not effective in controlling parasitemia or mortality of infected animals in the benznidazole control group [81].

In leishmaniasis, it was observed that silybin and its oxidized and prenylated derivatives have binding affinities to the recombinant cytosolic domain of the Pgp-like transporter of *Leishmania*. These compounds were able to reverse drug resistance in a *L. tropica* strain that overexpressed this transporter. Furthermore, treatment with cisplatin in combination with silybin reduced parasite load and increased Th1-type immune responses in animals infected with *L. donovani* [83].

The studies also demonstrated the antiparasitic activity of silybin and its derivatives against other important protozoa, such as *N. fowleri* and different species of *Acanthamoeba* [17]. These compounds showed activity with an IC_50_ below 25 µM and favorable selectivity indices, indicating their therapeutic potential against infections by these organisms [17].

## 7. Mechanism of Antibacterial Activity

The putative mechanisms by which silybin inhibits bacterial growth have been recently described. These mechanisms include the inhibition of efflux pumps, nucleic acid, protein synthesis, and biofilm formation, the reduction of virulence factors, and the induction of death, similar to apoptosis [9,26,27,33,34,36,38,39,40,41]. Furthermore, most of these studies mainly involved species of Gram-positive bacteria, with a predominance of MRSA. The main mechanisms of action of silybin are shown in Table 4.

### 7.1. Inhibition of Efflux Pumps

Efflux pumps are protein complexes present in bacterial membranes that are responsible for conferring resistance, as they function by expelling antimicrobial agents from the cell [26]. The quicker the efflux pump system expels the antibacterial agents crossing the membrane, the less direct contact there is between the bacteria and these agents. This reduces the bactericidal effect of distinct antimicrobials and contributes to pathogen resistance at the membrane level [86]. This is concerning because this mechanism can contribute to drug resistance in bacteria through the active removal of distinct classes of antibiotics [39].

Silybin acts as an inhibitor of efflux pumps in bacterial cells. Several studies have shown that the quinolone resistance protein NorA (*norA*) and quaternary ammonium resistance protein A/B (*qacA/B*) systems are the main efflux pumps of MRSA [26,27,33,34]. Silybin at a concentration of 1.25 µg/mL inhibited NorA, an efflux pump present in the MRSA membrane [33]. Similarly, in a study by Wang et al. [26], silybin reduced the expression of two NorA and AB pump efflux genes in MRSA. Corroborating these findings, Jung and Lee [27] also evidenced that silybin diminishes the activity of ABC pumps in *S. aureus*. Holasová et al. [34] demonstrated that flavolignanas such as silybin modulate the resistance to antibiotics and the virulence of *S. aureus*, affecting the corresponding efflux pumps such as ABC, MATE, and MFS. Recently, a study by Fekri Kohan et al. [39] showed that silybin reduces the expression of the AcrABZ-TolC efflux pump system in uropathogenic *E. coli*.

### 7.2. Inhibition of Nucleic Acids and Protein Synthesis

Bacterial nucleic acids, consisting of DNA and RNA, play essential roles in the maintenance and reproduction of bacterial cells. DNA molecules are responsible for storing, copying, and transmitting genetic information. RNA molecules, in turn, function as messengers to ensure adequate protein synthesis [86]. Lee et al. [41] found that silybin has inhibitory activity on nucleic acids, such as RNA, and on protein synthesis in *B. subtilis* and *S. epidermidis*. In addition, a recent study demonstrated that silybin can affect the fragmentation of DNA molecules in *E. coli* [36].

### 7.3. Inhibition of Biofilm Formation and Reduction of Virulence Factor Expression

Biofilm formation is a fully organized multistep process in which bacteria constantly communicate with each other. Furthermore, bacterial communication plays an essential role in bacterial life, since bacterial cells can detect and respond to autoinducers or other molecules, and accordingly adjust the production of virulence factors, bioluminescence, biofilm formation, and other factors. Thus, compounds able to interfere with bacterial communication have promising therapeutic potential in the field of bacterial virulence regulation [9,34]. Silybin affects pathways involved in bacterial quorum sensing; therefore, this compound is capable of preventing bacteria from adhering to the target tissue [34]. In the study by Holasová et al. [34], silybin was able to reduce bacterial communication and, in addition, was also able to inhibit the surface colonization of *S. aureus*. Hurtová et al. [9] proved that silybin A and silybin B are able to disrupt biofilm formation in *S. aureus* and *P. aeruginosa*, with an IC_50_ value of less than 100 µM. Furthermore, for the first time in the literature, these authors developed halogenated derivatives of silybin and found that they presented a superior inhibitory effect compared with the original compound, with an IC_50_ value below 10 µM. Despite presenting good activity against biofilm formation, none of the tested compounds were able to disrupt mature biofilms.

Another study showed that silybin inhibits biofilm formation in resistant *K. oxytoca* isolates through the reduction of some virulence factors, such as adhesins [40]. Omer et al. [40] demonstrated that from 100 mg/mL silybin was able to reduce the expression of the *fimA* and *mrkA* genes, which are responsible for bacterial adhesion and colonization and can mediate adhesion and biofilm formation. These results are also in agreement with those reported by Shen et al. [44] for Gram-positive isolates. Indeed, silybin was also able to reduce the expression of virulence genes of *S. suis* serotype 2 [44]. Silybin’s effects on the expression of virulence factors may be related to its effects on quorum sensing genes, which play an important role in the regulation of other biological factors, such as pathogenicity, biofilm, and secretion systems [9].

In a recent study, silybin was shown to downregulate the expression of the virulence genes *acrA*, *acrB*, and *tolC*, which encode efflux pumps, and upregulate the expression of genes encoding porins in uropathogenic *E. coli*. In this context, silybin upregulates the expression of *ompC* and *ompF* genes encoding porins (proteins that facilitate the entry of substances into the bacterial cell) [39]. In a more recent study, significant concentration-dependent inhibition of biofilm formation against *P. aeruginosa* (70.21%) and *K. pneumoniae* (71.02%) was reported for silybin at 30 μg/mL, and the greatest destruction of preformed biofilm was observed at 100 μg/mL against *P. aeruginosa* (89.74%) and *K. pneumoniae* (77.65%) in comparison with the individual bacterial control. Furthermore, a live/dead fluorescence assay for bacterial biofilms confirmed that 100 μg/mL silybin effectively inhibits biofilm formation by these pathogens [38].

### 7.4. Induction of Apoptosis-Like Death

**Figure 2:**
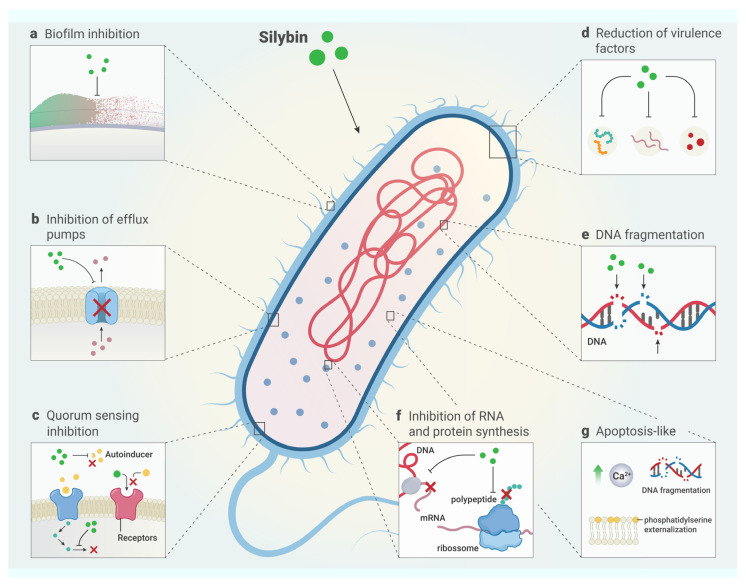
Antibacterial mechanism of silybin. Silybin exerts its antibacterial action through different mechanisms, including (**a**) inhibition of biofilm formation and biofilm formation, disrupting adherent bacterial communities; (**b**) inhibition of the expression of efflux pump genes, such as NorA, ABC and AcrABZ-TolC pumps, increasing the intracellular concentration of silybin; (**c**) inhibition of quorum sensing, limiting bacterial communication; (**d**) reduction of virulence factors, such as adhesins that are essential for the cell adhesion process; (**e**) DNA fragmentation, resulting in damage to the genetic material; (**f**) inhibition of RNA and protein synthesis; and (**g**) apoptosis-like death, promoting DNA fragmentation and cell death.

In recent studies, a new mechanism of prokaryotic cell death has been postulated, which is similar to the apoptotic cell death of eukaryotes and is called bacterial apoptosis-like death [87,88]. *E. coli* cells undergoing apoptosis-like death exhibit features of apoptosis, such as caspase-like protein activation, membrane depolarization, and exposure to phosphatidyldyserine [88,89]. DNA fragmentation is characteristic of apoptosis-like death. Although the exact mechanism has not been elucidated, it has been suggested that the RecA protein acts as a caspase in *E. coli* and fragments DNA strands. In a study developed by Lee and Lee [36], it was demonstrated that silybin caused the depolarization of the *E. coli* membrane and increased intracellular Ca^2+^ levels, consistent with bacterial apoptosis. In addition, cells treated with MIC and higher concentrations of silybin presented apoptotic characteristics, such as DNA fragmentation, exposure to phosphatidylserine, and expression of caspase-like protein. The mechanisms underlying the antibacterial actions of silybin are shown in Figure 2.

## 8. Mechanism of Antifungal Activity

The main mechanisms of silybin’s antifungal actions, including the inhibition of biofilm formation and induction of apoptosis, have not yet been well elucidated and explained in the scientific literature [11,47]. Recent studies describe different mechanisms by which silybin inhibits *C.* *albicans*, including from mitochondrial changes to plasma membrane damage. These are shown in Table 5.

**Figure 3:**
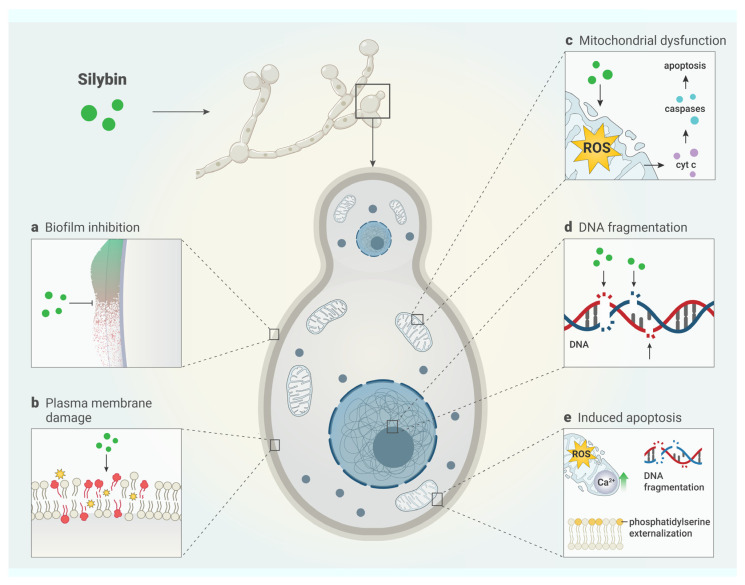
Antifungal mechanism of silybin. Silybin exerts its antifungal action through different mechanisms, including (**a**) inhibition of biofilm formation, disrupting adherent bacterial communities; (**b**) damage to the plasma membrane, causing cell rupture; (**c**) mitochondrial alterations, generating an increase in reactive oxygen species (ROS), intensifying oxidative stress; (**d**) DNA fragmentation, resulting in damage to the genetic material; and (**e**) induction of apoptosis, promoting cell disintegration and death.

Yun and Lee (2016) [47] demonstrated in a study that silybin has the potential to induce cell apoptosis in *C. albicans* yeast, mainly through mitochondrial Ca^2+^ signaling. In addition, it has been demonstrated that mitochondrial rupture generates the release of cytochrome C into the cytosol, activating the expression of caspase-like proteins, which trigger programmed cell death. The increase in reactive oxygen species in the mitochondria and cytosol leads to phosphatidylserine exposure in the cell membrane and DNA damage. To better understand the mechanisms involved in the inhibition of *C.* *albicans*, Yun and Lee (2017) [11] also demonstrated that silybin triggers plasma membrane damage as well as the inhibition of biofilm formation in its initial phase. Silybin also inhibits the hyphal extension of *C. albicans*, thus negatively influencing the formation of the biofilm structure. In preformed and mature biofilms, silybin showed almost no effects due to the strength of the established structure. However, early-stage biofilm was affected by silybin concentrations higher than 100 µg/mL; the biofilm did not actively proliferate, and its metabolic activity decreased. The mechanisms underlying the antifungal actions of silybin are shown in Figure 3.

## 9. Mechanism of Antiviral Activity

The relevant experiments describing the mechanisms of the antiviral action of silybin are presented in Table 6. The primary mechanism of its antiviral activity includes blocking essential viral enzymes, such as RNA polymerase, proteases, and viral capsid protein binding.

## 10. The Combined Use of Silybin with Other Antimicrobial Drugs

When used in combination with antibiotics, some phenolic compounds, including silybin, are capable of enhancing their effects and, in some cases, reversing bacterial resistance to specific antibiotics [8]. Interestingly, under normal conditions, this type of mechanism of action, usually synergistic, offers a great advantage because it is unlikely to represent selective pressure for the development of resistance in microorganisms [8]. Furthermore, it is important to highlight that bioactive compounds can interact in different ways, and these interactions can be classified as synergistic, additive, or antagonistic [90].

Considering the wide range of antimicrobial benefits of silybin, many studies have shown it enhances the effects of other antimicrobial drugs. In Gram-negative bacteria, silybin, when combined with antibiotics used in the clinics such as chloramphenicol, kanamycin [27], amikacin, and ciprofloxacin [14], showed a synergistic effect in the inhibition of *P. aeruginosa* strains. However, when combined with gentamicin and imipenem, silybin demonstrated an antagonistic effect against *P. aeruginosa* [14]. Similarly, when combined with gentamicin, it demonstrated a synergistic effect against *E. coli* [14]. In a recent study, silybin also demonstrated a synergistic effect when combined with ciprofloxacin against clinical isolates of uropathogenic *E. coli* [39].

Other studies have also revealed an interaction between silybin and antimicrobials used against Gram-positive bacteria. Silybin demonstrated a synergistic effect when combined with oxacillin and ampicillin against MRSA strains [43], as well as synergistic and additive effects when combined with gentamicin and ampicillin against oral pathogens, including *S. mutans* and *P. gingivalis* [35]. Furthermore, when combined with amikacin, kanamycin, gentamicin, and imipenem, silybin showed synergistic effects against *S. aureus* [14,30]; it also showed synergistic effects with ampicillin against MRSA and MSSA [15]. Additionally, a recent study demonstrated that silybin demonstrated an antagonistic effect when combined with nystatin and no effect when combined with mebendazole against *C. albicans*, *C. krusei*, and *C. tropicalis* [14]. In addition to the combined treatment of silybin with antimicrobials used against bacterial and fungal infections, some researchers have demonstrated in their studies that treatment with cisplatin in combination with silybin resulted in a reduction in parasite load and an increase in Th1-type immune responses in animals infected with *L. donovani* [83].

## 11. Bioavailability of Silybin

Although silybin was reported to have significant antimicrobial activity, it possesses relatively low oral bioavailability. Silybin has low solubility in water (less than 50 μg/mL) due to its highly hydrophobic and non-ionizable chemical structure, which greatly influences its bioavailability. However, the solubility of this compound increases significantly in the presence of certain organic solvents, such as transcutol (350.1 mg/mL), ethanol (225.2 mg/mL), polysorbate 20 (131.3 mg/mL), and glycerin (33.2 mg/mL) [21]. This stark contrast in solubility between aqueous and organic environments underscores the importance of formulation strategies for improving silybin bioavailability.

When orally administered, silybin is rapidly absorbed, reaching its maximum plasma concentration (tmax) at 2–4 h, with a half-life of 6 h. After administration, only 20–50% of the silybin is absorbed from the gastrointestinal (GI) tract and metabolized in the liver [29]. It is reported that silybin B is absorbed more rapidly compared with silybin A after the intragastric administration of each diastereoisomer of silybin in rats [91]. Human and animal studies have suggested that the absorbed silybin undergoes rapid and extensive phase II conjugative metabolism with primary biliary excretion, resulting in a short half-life and low systemic exposure following oral administration [92,93,94]. The main metabolites detected after the oral administration of silybin in human plasma are glucuronides (about 55%) and sulfates (about 28%), which are catalyzed by the enzymes UDP-glucuronyltransferase and sulfatransferase [95,96]. Interestingly, silybin metabolism exhibits stereoselectivity, adding another layer of complexity to its pharmacokinetic profile. Studies in rats showed that silybin B, a stereoisomer of silybin, is absorbed more rapidly than silybin A [91]. This differential absorption rate between stereoisomers suggests that the spatial configuration of the molecule plays a crucial role in its interaction with the absorption mechanisms in the gastrointestinal tract. Consequently, the main challenges in the clinical use of silybin are its low bioavailability, rapid conjugation, and extensive biliary excretion. These factors have hindered its application as a pharmaceutical product [97].

## 12. Silybin and Nanotechnology

Nanotechnology can overcome some limitations regarding bioavailability, providing favorable characteristics to promote the healing effect of therapeutic molecules of natural origin, including silybin [97]. Therefore, through different approaches using nanotechnology, studies have been carried out in order to increase the bioavailability of silybin and, at the same time, enhance its antimicrobial therapeutic activity. Recently, it was reported that silybin nanoparticles demonstrated higher antimicrobial activity against a wide range of microorganisms of clinical interest, including bacterial strains (*B. subtilis*, *S. aureus*, *E. coli*, *K. pneumoniae*, *P. aeruginosa*) [29,32,37,38,39], fungi (*C. albicans*, *C. glabrata*) [29] and viruses (HCV) [69], surpassing its activity in the pure form. In the study developed by Sahibzada et al. [29], nanoparticles prepared by antisolvent precipitation using the syringe pump (APSP) and evaporative precipitation of nanosuspension (EPN) methods exhibited strong antibacterial activity against *B. subtilis* and *S. aureus*. However, even at the highest concentrations tested, they did not show activity against Gram-negative bacteria such as *E. coli* and *P. aeruginosa*. The antifungal action of these nanoparticles against strains of *C.* *albicans* and *C. glabrata* was also demonstrated, with the EPN method showing greater inhibitory activity. Islan et al. [37] developed silybin-functionalized gold nanoparticles (S-AuNPs) to evaluate their activity against clinical pathogens related to nosocomial infections, including *E. coli* and *P. aeruginosa*. The live/dead fluorescent assay confirmed that S-AuNPs could kill bacteria with significant cell damage after only 30 min of exposure. Furthermore, transmission electron microscopy (TEM) imaging demonstrated that S-AuNPs could strongly interact with the surfaces of these bacteria, leading to cell lysis. In another study, Vimalraj et al. [32] demonstrated that zinc–silybin complexes exhibited higher inhibitory activity against *S. aureus* and *E. coli* when compared with pure silybin. In a recent study, silybin-loaded chitosan-coated silver nanoparticles (S-C@AgNPs) were synthesized to evaluate their antimicrobial potential against resistant nosocomial pathogens, including *E. coli*, *P. aeruginosa*, *K. pneumoniae*, and *E. faecalis* [38]. It was demonstrated that silybin has potential antibiofilm activity against *K. pneumoniae* and *P. aeruginosa*, preventing the formation of biofilms at concentrations ≤ 100 µg/mL.

Fekri Kohan et al. [39] recently evaluated the effects of silybin loaded polymers (SPNs) in combination with ciprofloxacin or meropenem in *E. coli* isolates and, through quantitative analysis, suggested that silybin potentially increases antibiotic susceptibility in resistant isolates through multiple mechanisms. These mechanisms include the downregulation of efflux pump genes and upregulation of porins, culminating in increased antibiotic uptake by bacterial cells, thereby enhancing antibiotic-mediated bacterial cell death. In addition to studies on bacteria and fungi, it was also demonstrated that silybin nanoparticles based on hydrolysable carriers have a stronger antiviral effect against HCV infection than those in their pure form [69]. Additionally, other studies have already reported that the application of nanotechnology favors an increase in the bioavailability and the hepatoprotective effect of silybin [46]. Likewise, pretreatment with silybin NPs protects against drug-induced hepatotoxicity [97,98].

## 13. Conclusions and Future Directions

Silybin has demonstrated good antimicrobial activities against various bacterial, fungal, viral, and parasitic pathogens. However, the collective impact of some factors, such as its poor water solubility, limited absorption, rapid metabolism, and extensive biliary excretion, presents significant hurdles for the development of silybin as an effective pharmaceutical product. These challenges require the development and implementation of innovative strategies to overcome the low bioavailability of silybin. Potential approaches may include the development of novel formulations to enhance solubility and absorption, the use of drug delivery systems to protect silybin from rapid metabolism, or the design of prodrug forms that can bypass extensive first-pass metabolism.

Ongoing investigations focused on enhancing the solubility and bioavailability of silybin have suggested its potential as a novel therapeutic agent for the prevention and treatment of infectious diseases. More in vivo studies are essential to demonstrate its effectiveness in animal models and assess its performance in clinical trials.

Further investigation into the potential synergistic effects of silybin with existing antibiotics is needed to open new avenues for combination therapies, potentially enhancing treatment efficacy while minimizing the development of antimicrobial resistance. This approach could be particularly valuable in addressing the growing concern regarding antibiotic-resistant pathogens, as combining silybin with conventional antibiotics may allow for lower dosages of both compounds while maintaining or even improving therapeutic outcomes. Such combinations could potentially reduce side effects associated with high antibiotic doses and slow the emergence of resistant strains.

Additionally, exploring the structure–activity relationships of silybin derivatives may lead to the development of more potent and targeted antimicrobial compounds. Elucidation of the molecular mechanisms underlying the antimicrobial activities of silybin could provide valuable insights into the design of novel therapeutic strategies against infectious diseases. Understanding how silybin interacts with bacterial cells at the molecular level, including its effects on cell membranes, metabolic pathways, or gene expression, could reveal new targets for antimicrobial drug development. This knowledge can also help predict potential resistance mechanisms and guide the design of compounds that can overcome or circumvent these resistance pathways.

In addition, exploring the potential of silybin and its derivatives in treating biofilm-associated infections could address a significant challenge in current antimicrobial therapy. Many chronic and recurrent infections are associated with bacterial biofilms, which are difficult to eradicate using conventional antibiotics. If silybin demonstrates antibiofilm activities or the ability to penetrate existing biofilms, it could provide a valuable tool for treating these persistent infections, potentially reducing the need for long-term antibiotic use and decreasing the risk of antibiotic resistance development.

## Figures and Tables

**Table 1:** Antibacterial activity of silybin.

Species *	Method	Activity **	Location	References
Gram-negative bacteria				
*Acinetobacter baumannii*	Microdilution	MIC: 8–64 µg/mL	Turkey	[28]
*Aggregatibacter* *actinomycetemcomitans*	Microdilution Checkerboard Time kill curve	MIC: 1.6 µg/mL	Republic of Korea	[35]
*Escherichia coli*	Microdilution Checkerboard	MIC: 20 µg/mL	Republic of Korea	[27]
	Microdilution	MIC: 8–64 µg/mL	Turkey	[28]
	Microdilution Checkerboard	MIC: 64 µg/mL	Brazil	[14]
	Microdilution Time kill	MIC: 40 µg/mL	Republic of Korea	[36]
	Microdilution	MIC: >512 µg/mL	Pakistan	[29]
	Microdilution	MIC: 1.25 µM	Iran	[31]
	Microdilution Disc diffusion	MIC: 1–10 μg/mL IZ: 7–8 mm	India	[32]
	Microdilution	MIC: 5.6 μg/mL	Argentina	[37]
	Microdilution Disc diffusion	MIC: 1.55–3.12 µg/mL IZ: 8–12 mm	India	[38]
	Microdilution Checkerboard	MIC: 128–512 μg/mL	Iran	[39]
*Fusobacterium nucleatum*	Microdilution Checkerboard Time kill curve	MIC: 3.2 µg/mL	Republic of Korea	[35]
*Helicobacter pylori*	Microdilution	MIC: 256 µg/mL	Brazil	[12]
*Klebsiella oxytoca*, *Klebsiella pneumoniae*	Microdilution	MIC: 8–64 µg/mL	Turkey	[28]
	Microdilution Biofilm formation	MIC: 100–500 mg/mL	Iraq	[40]
	Microdilution Disc diffusion	MIC: 1.55–6.25 µg/mL IZ: 10–15 mm	India	[38]
*Porphyromonas gingivalis*	Microdilution Checkerboard Time kill curve	MIC: 0.4 µg/mL	Republic of Korea	[35]
*Prevotella intermedia*	Microdilution Checkerboard Time kill curve	MIC: 1.6 µg/mL	Republic of Korea	[35]
*Proteus mirabilis*	Microdilution	MIC: 8–64 µg/mL	Turkey	[28]
*Pseudomonas aeruginosa*	Microdilution Checkerboard	MIC: 10–20 µg/mL	Republic of Korea	[27]
	Microdilution	MIC: 4–32 µg/mL	Turkey	[28]
	Microdilution Checkerboard	MIC: 1.024 µg/mL	Brazil	[14]
	Microdilution	MIC: >512 µg/mL	Pakistan	[29]
	Microdilution	MIC: 0.625 µg/mL	Iran	[31]
	Microdilution	MIC: 11.2 µg/mL	Argentina	[37]
	Biofilm formation	Active in concentrations < 10 μM	Czech Republic	[9]
	Microdilution Disc diffusion	MIC: 1.55–6.25 µg/mL IZ: 11–15 mm	India	[38]
*Salmonella typhi*	Microdilution	MIC: 0.312 µg/mL	Iran	[31]
*Vibrio campbellii*	Quorum Sensing Inhibition	Active in concentrations < 10 μM	Czech Republic	[9]
Gram-positive bacteria				
*Bacillus subtilis*	Microdilution	IC_50_: 11.8 μg/mL	Republic of Korea	[41]
	Microdilution	MIC:16 μg/mL	Pakistan	[29]
*Corynebacterium xerosis*	Microdilution	MIC: 1.25 μg/mL	Iran	[31]
*Enterococcus faecalis*, *Enterococcus faecium*	Microdilution Checkerboard	MIC: >20 µg/mL	Republic of Korea	[27]
	Microdilution	MIC: 2–64 µg/mL	Turkey	[28]
	Microdilution Disc diffusion	MIC: 1.55 µg/mL IZ: 7–22 mm	India	[38]
*Mycobacterium tuberculosis*	Microdilution Colony forming unit assay	MIC: 50–400 μM	Mexico	[42]
*Staphylococcus aureus*, *Staphylococcus epidermidis*, MRSA, MSSA	Microdilution	MIC: 1.25 µg/mL	United States of America	[33]
	Microdilution	IC_50_: 15.7 µg/mL	Korea	[41]
	Microdilution Checkerboard	MIC: 1.25–10 µg/mL	Republic of Korea	[27]
	Microdilution Checkerboard Time kill curve	MIC: 2–8 µg/mL	South Korea	[43]
	Microdilution	MIC: 2–64 µg/mL	Turkey	[28]
	Microdilution Checkerboard	MIC: 1.024 µg/mL	Brazil	[14]
	Microdilution	MIC: 32 µg/mL	Pakistan	[29]
	Colony forming unit assay	Active in concentrations of 400 µM	China	[30]
	Microdilution	MIC: 0.312 µg/mL	Iran	[31]
	Microdilution Disc diffusion	MIC: 1–10 µg/mL IZ: 7–8 mm	India	[32]
	Double dilution	MIC: 32 μg/mL	China	[26]
	Microdilution Checkerboard	MIC: 62.5–250 μg/mL	Saudi Arabia	[15]
	Efflux pump inhibition Quorum Sensing Inhibition	MIC: 5–40 μM	Czech Republic	[34]
	Biofilm inhibition	Active in concentrations < 10 μM	Czech Republic	[9]
*Streptococcus anginosus*, *Streptococcus criceti*, *Streptococcus gordonii*, *Streptococcus mutans*, *Streptococcus ratti*, *Streptococcus sanguinis*, *Streptococcus sobrinus*, *Streptococcus suis*	Microdilution Checkerboard Time kill curve	MIC: 0.1–0.8 μg/mL	Republic of Korea	[35]
	Microdilution	MIC: >1.024 μg/mL	China	[44]

* MRSA: Methicillin-resistant *Staphylococcus aureus*; MSSA: Methicillin-susceptible *Staphylococcus aureus*. ** IZ: Inhibition Zone; MIC: Minimum Inhibitory Concentration; IC_50_: half maximal inhibitory concentration.

**Table 2:** Antifungal activity of silybin.

Gender	Species	Method	Activity *	Location	References
*Aspergillus*	*A. flavus*	Double dilution	MIC: 20 µM	Republic of Korea	[47]
*Candida*	*C. albicans*, *C. glabrata*, *C. krusei*, *C. parapsilosis*, *C. tropicalis*	Microdilution	MIC: 4–8 µg/mL	Turkey	[28]
		Microdilution Checkerboard	MIC: 1.024 µg/mL	Brazil	[14]
		Double dilution	MIC: 20–40 µM	Republic of Korea	[47]
		Microdilution	MIC: 64–512 µg/mL	Pakistan	[29]
		Biofilm formation	Active in concentrations above 100 μM	Republic of Korea	[11]
		Disc diffusion	Active in concentrations of 15, 20, 25 mg/mL	India	[48]
*Malassezia*	*M. furfur*	Double dilution	MIC: 40 µM	Republic of Korea	[47]
			Active in concentrations of 15, 20, 25 mg/mL	India	[48]
*Trichosporon*	*T. beigelii*	Double dilution	MIC: 20–40 µM	Republic of Korea	[47]

* MIC: Minimum Inhibitory Concentration.

**Table 3:** Antiparasitic activity of silybin.

Protozoan	Identification	Forms of Development	Main Conclusions	Location	References
*Trypanosoma brucei*	STIB 900	Trypomastigote forms of the bloodstream	(i) potent and non-competitive inhibition of TbAT1 mediated adenosine transport in yeast; (ii) inhibition of melarsen-induced lysis of bloodstream trypanosomes with IC_50_ ± SEM de 6.0 ± 0.0 × 10^2^.	USA	[80]
*Leishmania tropica*	DNM-R150	Promastigotes	silybin and, mainly, its oxidized and prenylated derivatives show high binding affinity to the recombinant cytosolic domain of the *Leishmania* Pgp-like transporter and reverse the MDR of a *L. tropica* strain that overexpresses the transporter.	Spain	[82]
*Leishmania donovani*	MHOM/IN/80/Dd8	Promastigotes	reduction in parasite load, increase in Th1-type immune responses and normalization of several biochemical parameters occurred in animals treated with cisplatin in combination with silybin.	India	[83]
*Mesocestoides vogae*	-	-	silybin and its derivative 2,3-dehydrosilybin suppressed mitochondrial functions and energy stores, inducing a physiological imbalance, while 2,3-dehydrosilybin exhibited a direct larvicidal effect due to damage to the tegument and complete disruption of larval physiology and metabolism.	Czech Republic	[85]
*Leishmania* *infantum*	Li UCM9 (M/CAN/ES/2001/UCM9)	Promastigotes	silybin did not cause any inhibition of *Leishmania* promastigotes; however, its derivative dehydrosilybin A significantly inhibited Li promastigotes with an approximate IC_50_ of 90.23 µM.	Spain	[84]
*Leishmania* *donovani*	(MHOM/SD/43/124)	Promastigotes	there was a reduction of more than ≥30% (120 µM).	Spain	[84]
*Trypanosoma cruzi*	Strain Y	Epimastigotes	inhibition of parasite growth (25 µM).	Brazil	[81]
*Trypanosoma cruzi*	Strain Y	Amastigotes	(i) silybin presented IC_50_ and selectivity index of 79.81 μM and 3.13, respectively; (ii) the combination of silybin + benznidazole presented inhibition of 91.44%; (iii) monotherapy with silybin was not able to control parasitemia/mortality of the animals.	Brazil	[81]
*Naegleria fowleri*	ATCC 30215	Trophozoites	activity with IC_50_ ± SD < 25 µM with selectivity index equal to 4.13 μM.	Republic of Korea	[17]
*Acanthamoeba castellanii*	ATCC 30868	Trophozoites	activity with IC_50_ ± SD < 26 µM with selectivity index equal to 4.08 μM.	Republic of Korea	[17]
*Acanthamoeba polyphaga*	ATCC 30461	Trophozoites	activity with IC_50_ ± SD < 16 µM with selectivity index equal to 6.31 μM.	Republic of Korea	[17]

ATCC: American Type Culture Collection; MDR: multidrug resistance.

**Table 4:** Antibacterial mechanism of silybin.

Mechanism of Action	Name of the Bacteria	Detailed Mechanisms of Action	References
Inhibition of efflux pump	MRSA	It acts by inhibiting the NorA efflux pump.	[33]
	MRSA	It acts by inhibiting the ABC efflux pump.	[27]
	MRSA	Reduced expression of the quinolone resistance protein NorA (*norA*) and quaternary ammonium resistance protein A/B (*qacA/B*) efflux genes.	[26]
	MRSA	Antibiotic-induced reduction of gene expression of representative efflux pumps belonging to the major facilitator (MFS), multiple and toxic compound extrusion (MATE), and ATP-binding cassette (ABC) families.	[34]
	*Escherichia coli*	Downregulation of the efflux pump genes AcrAB-TolC and upregulation of the porin genes *ompC* and *ompF* in combination with ciprofloxacin at the transcriptional level.	[39]
Inhibition of nucleic acid and protein synthesis	*Bacillus subtilis*, *Staphylococcus epidermidis*	It acts by inhibiting the synthesis of RNA and proteins.	[41]
	*Escherichia coli*	It acts on DNA fragmentation.	[36]
Biofilm inhibition and quorum sensing	MRSA	Reduction of virulence factors, namely bacterial communication between cells and cell adhesion to the surface.	[34]
	*Staphylococcus aureus*, *Pseudomonas aeruginosa*, *Vibrio campbellii*	Reduction of virulence factors, namely cell adhesion to the surface and communication between bacteria. Prevention of biofilm formation.	[9]
	*Klebsiella oxytoca*	Reduction of virulence factors.	[40]
	*Escherichia coli*, *Pseudomonas aeruginosa*	Prevention of biofilm formation and inhibition of formed biofilm.	[38]
	*Escherichia coli*	Notable reduction in bacterial growth and biofilm formation in ciprofloxacin-resistant isolates.	[39]
Induction of apoptosis-like death	*Escherichia coli*	Induction of apoptosis-like cell death mediated by membrane depolarization with Ca^2+^ signaling. Apoptosis induced by exposure to phosphatidylserine and activation of caspase-like proteins.	[36]

MRSA: Methicillin-resistant *Staphylococcus aureus*.

**Table 5:** Antifungal mechanism of silybin.

Fungus Name	Mechanism of Action	References
*Candida albicans*	Induction of yeast apoptosis mediated by mitochondrial Ca^2+^ signaling.	[47]
	Mitochondrial dysfunction due to excess reactive oxygen species.	
	Induced apoptosis caused mitochondrial membrane depolarization, cytochrome C release, caspase-like protein activation, phosphatidylserine exposure, and DNA damage.	
	Apoptosis via oxidative stress increased by 24.17% compared to untreated cells.	
	Damage to the plasma membrane occurs and inhibits biofilm development in its initial phase.	[11]

**Table 6:** Antiviral mechanism of silybin.

Virus Name	Mechanism of Action	Location	References
Human enterovirus 68 (EV68)	Inhibition of ataxia telangiectasia mutated (ATM) and DNA-dependent protein kinase (DNA-PK).	China	[75]
Chikungunya virus	Interference with viral replication inhibition of viral attachment and entry and microneutralization.	India	[74]
Hepatitis B virus (HBV)	Blockade of clathrin-mediated endocytosis.	Japan	[49]
Hepatitis C virus (HCV)	Inhibition of the function of the RNA-dependent RNA polymerase NS5B.	France	[51]
Hepatitis C virus (HCV)	Inhibited innate inflammatory and antiviral signaling from NF-κB and IFN-κB promoters.	USA	[54]
	Inhibited expression of tumor necrosis factor alpha in human peripheral blood mononuclear cells stimulated with anti-CD3 and NF-κB-dependent transcription.	USA	[56]
	Inhibits the initial stages of infection by affecting the endosomal trafficking of virions.	France	[57]
	Inhibition of RNA replication silybin may target an interaction between NS4B and NS3/4A.	Germany	[59]
	Capsid protein binding.	India	[61]
	Inhibition of oxidative stress.	Taiwan	[69]
Human immunodeficiency virus type 1 (HIV-1)	Disruption of T cell metabolism in vitro; blockade of T cell infection by HIV.	USA	[71]
Influenza A virus (IAV)	S0 and S3 inhibited IAV replication and disrupted Atg5-Atg12/Atg16L complex formation.	China	[73]
Severe acute respiratory syndrome coronavirus 2 virus (SARS-CoV-2)	Inhibition of STAT3 and RNA-dependent RNA polymerase (RdRp).	Spain	[76]
	Inhibition of spike protein and RNA-dependent RNA polymerase.	Italy	[78]
	inhibition of SARS-CoV-2 main protease (M^pro^).	Italy	[77]
	Inhibition of spike protein (S), major protease (M^pro^), RNA-dependent RNA polymerase (RdRp).	United Arab Emirates	[79]
	Inhibition of viral entry, inhibition of viral replication and regulation of the immune response.	USA	[16]

## Data Availability

No new data were created or analyzed in this study.

## References

[1] Ayobami O., Brinkwirth S., Eckmanns T., Markwart R. (2022). Antibiotic resistance in hospital-acquired ESKAPE-E infections in low-and lower-middle-income countries: A systematic review and meta-analysis. Emerg. Microbes Infect..

[2] Getahun Y.A., Ali D.A., Taye B.W., Alemayehu Y.A. (2022). Multidrug-resistant microbial therapy using antimicrobial peptides and the CRISPR/Cas9 system. Vet. Med. Res. Rep..

[3] Dar M.A., Gul R., Karuppiah P., Al-Dhabi N.A., Alfadda A.A. (2022). Antibacterial activity of cerium oxide nanoparticles against ESKAPE pathogens. Crystals.

[4] Velazquez-Meza M.E., Galarde-López M., Carrillo-Quiróz B., Alpuche-Aranda C.M. (2022). Antimicrobial resistance: One health approach. Vet. World.

[5] Zhu Y., Huang W.E., Yang Q. (2022). Clinical perspective of antimicrobial resistance in bacteria. Infect. Drug Resist..

[6] Monteiro-Neto V., de Souza C.D., Gonzaga L.F., da Silveira B.C., Sousa N.C., Pontes J.P., Santos D.M., Martins W.C., Pessoa J.F., Carvalho Junior A.R. (2020). Cuminaldehyde potentiates the antimicrobial actions of ciprofloxacin against *Staphylococcus aureus* and *Escherichia coli*. PLoS ONE.

[7] Takó M., Kerekes E.B., Zambrano C., Kotogán A., Papp T., Krisch J., Vágvölgyi C. (2020). Plant phenolics and phenolic-enriched extracts as antimicrobial agents against food-contaminating microorganisms. Antioxidants.

[8] Donadio G., Mensitieri F., Santoro V., Parisi V., Bellone M.L., De Tommasi N., Izzo V., Dal Piaz F. (2021). Interactions with microbial proteins driving the antibacterial activity of flavonoids. Pharmaceutics.

[9] Hurtová M., Káňová K., Dobiasová S., Holasová K., Čáková D., Hoang L., Biedermann D., Kuzma M., Cvačka J., Křen V. (2022). Selectively halogenated flavonolignans—Preparation and antibacterial activity. Int. J. Mol. Sci..

[10] Monti D., Gažák R., Marhol P., Biedermann D., Purchartová K., Fedrigo M., Riva S., Křen V. (2010). Enzymatic kinetic resolution of silybin diastereoisomers. J. Nat. Prod..

[11] Yun D.G., Lee D.G. (2017). Assessment of silibinin as a potential antifungal agent and investigation of its mechanism of action. IUBMB Life.

[12] Bittencourt M.L.F., Rodrigues R.P., Kitagawa R.R., Gonçalves R.d.C.R. (2020). The gastroprotective potential of silibinin against *Helicobacter pylori* infection and gastric tumor cells. Life Sci..

[13] Verdura S., Cuyàs E., Ruiz-Torres V., Micol V., Joven J., Bosch-Barrera J., Menendez J.A. (2021). Lung cancer management with silibinin: A historical and translational perspective. Pharmaceuticals.

[14] Rakelly de Oliveira D., Relison Tintino S., Morais Braga M.F.B., Boligon A.A., Linde Athayde M., Douglas Melo Coutinho H., de Menezes I.R.A., Fachinetto R. (2015). In vitro antimicrobial and modulatory activity of the natural products silymarin and silibinin. BioMed Res. Int..

[15] Alhadrami H.A., Hamed A.A., Hassan H.M., Belbahri L., Rateb M.E., Sayed A.M. (2020). Flavonoids as potential anti-MRSA agents through modulation of PBP2a: A computational and experimental study. Antibiotics.

[16] Zhang C., Sui Y., Liu S., Yang M. (2023). Anti-viral activity of bioactive molecules of silymarin against COVID-19 via in silico studies. Pharmaceuticals.

[17] Lê H.G., Võ T.C., Kang J.-M., Nguyễn T.H., Hwang B.-S., Oh Y.-T., Na B.-K. (2023). Antiamoebic activities of flavonoids against pathogenic free-living amoebae, *Naegleria fowleri* and *Acanthamoeba* species. Parasites Hosts Dis..

[18] Fallah M., Davoodvandi A., Nikmanzar S., Aghili S., Mirazimi S.M.A., Aschner M., Rashidian A., Hamblin M.R., Chamanara M., Naghsh N. (2021). Silymarin (milk thistle extract) as a therapeutic agent in gastrointestinal cancer. Biomed. Pharmacother..

[19] Pelter A., Hänsel R. (1968). The structure of silybin (silybum substance E6), the first flavonolignan. Tetrahedron Lett..

[20] Pelter A., Hansel R. (1975). Structure of silybin. 1. Degradative experiments. Chem. Berichte-Recl..

[21] Bijak M. (2017). Silybin, a major bioactive component of milk thistle (*Silybum marianum* L. Gaernt.)—Chemistry, bioavailability, and metabolism. Molecules.

[22] Biedermann D., Vavříková E., Cvak L., Křen V. (2014). Chemistry of silybin. Nat. Prod. Rep..

[23] Křen V. (2021). Chirality Matters: Biological activity of optically pure silybin and its congeners. Int. J. Mol. Sci..

[24] Hao Y., Wei Z., Wang Z., Li G., Yao Y., Dun B. (2021). Biotransformation of flavonoids improves antimicrobial and anti-breast cancer activities in vitro. Foods.

[25] Hanci H., Igan H. (2023). Antimicrobial synergistic effects of apigenin, (-)-epigallocatechin-3-gallate, myricetin and luteolin in combination with some antibiotics. Ann. Agric. Environ. Med..

[26] Wang D., Xie K., Zou D., Meng M., Xie M. (2018). Inhibitory effects of silybin on the efflux pump of methicillin-resistant *Staphylococcus aureus*. Mol. Med. Rep..

[27] Jung H.J., Lee D.G. (2008). Synergistic antibacterial effect between silybin and N, N′-dicyclohexylcarbodiimide in clinical *Pseudomonas aeruginosa* isolates. J. Microbiol..

[28] Özçelik B., Kartal M., Orhan I. (2011). Cytotoxicity, antiviral and antimicrobial activities of alkaloids, flavonoids, and phenolic acids. Pharm. Biol..

[29] Sahibzada M.U.K., Sadiq A., Khan S., Faidah H.S., Naseemullah, Khurram M., Amin M.U., Haseeb A. (2017). Fabrication, characterization and in vitro evaluation of silibinin nanoparticles: An attempt to enhance its oral bioavailability. Drug Des. Dev. Ther..

[30] Cai J.-Y., Li J., Hou Y.-N., Ma K., Yao G.-D., Liu W.-W., Hayashi T., Itoh K., Tashiro S.-i., Onodera S. (2018). Concentration-dependent dual effects of silibinin on kanamycin-induced cells death in *Staphylococcus aureus*. Biomed. Pharmacother..

[31] Rahimifard M., Moini-Nodeh S., Niaz K., Baeeri M., Jamalifar H., Abdollahi M. (2018). Improvement of the functionality of pancreatic Langerhans islets via reduction of bacterial contamination and apoptosis using phenolic compounds. Iran. J. Basic Med. Sci..

[32] Vimalraj S., Rajalakshmi S., Saravanan S., Preeth D.R., Vasanthi R.L., Shairam M., Chatterjee S. (2018). Synthesis and characterization of zinc-silibinin complexes: A potential bioactive compound with angiogenic, and antibacterial activity for bone tissue engineering. Colloids Surf. B Biointerfaces.

[33] Stermitz F.R., Tawara-Matsuda J., Lorenz P., Mueller P., Zenewicz L., Lewis K. (2000). 5′-Methoxyhydnocarpin-D and Pheophorbide a: Berberis species components that potentiate Berberine growth inhibition of resistant *Staphylococcus aureus*. J. Nat. Prod..

[34] Holasová K., Křížkovská B., Hoang L., Dobiasova S., Lipov J., Macek T., Křen V., Valentová K., Ruml T., Viktorova J. (2022). Flavonolignans from silymarin modulate antibiotic resistance and virulence in *Staphylococcus aureus*. Biomed. Pharmacother..

[35] Lee Y.-S., Jang K.-A., Cha J.-D. (2012). Synergistic antibacterial effect between silibinin and antibiotics in oral bacteria. Biomed. Res. Int..

[36] Lee B., Lee D.G. (2017). Reactive oxygen species depletion by silibinin stimulates apoptosis-like death in *Escherichia coli*. J. Microbiol. Biotechnol..

[37] Islan G.A., Das S., Cacicedo M.L., Halder A., Mukherjee A., Cuestas M.L., Roy P., Castro G.R., Mukherjee A. (2019). Silybin-conjugated gold nanoparticles for antimicrobial chemotherapy against Gram-negative bacteria. J. Drug Deliv. Sci. Technol..

[38] Chand U., Kushawaha P.K. (2024). Silibinin-loaded chitosan-capped silver nanoparticles exhibit potent antimicrobial, antibiofilm, and anti-inflammatory activity against drug-resistant nosocomial pathogens. J. Biomater. Sci. Polym. Ed..

[39] Fekri Kohan S., Nouhi Kararoudi A., Bazgosha M., Adelifar S., Hafezolghorani Esfahani A., Ghaderi Barmi F., Kouchakinejad R., Barzegari E., Shahriarinour M., Ranji N. (2024). Determining the potential targets of silybin by molecular docking and its antibacterial functions on efflux pumps and porins in uropathogenic *E. coli*. Int. Microbiol..

[40] Omer F.H., Al-Khafaji N.S., Al-Alaq F.T., Al-Dahmoshi H.O., Memariani M., Saki M. (2022). Synergistic effects of silybin and curcumin on virulence and carbapenemase genes expression in multidrug resistant *Klebsiella oxytoca*. BMC Res. Notes.

[41] Lee D.G., Kim H.K., Park Y., Park S.-C., Woo E.-R., Jeong H.G., Hahm K.-S. (2003). Gram-positive bacteria specific properties of silybin derived from *Silybum marianum*. Arch. Pharmacal Res..

[42] Rodríguez-Flores E.M., Mata-Espinosa D., Barrios-Payan J., Marquina-Castillo B., Castañón-Arreola M., Hernández-Pando R. (2019). A significant therapeutic effect of silymarin administered alone, or in combination with chemotherapy, in experimental pulmonary tuberculosis caused by drug-sensitive or drug-resistant strains: In vitro and in vivo studies. PLoS ONE.

[43] Kang H.K., Kim H.Y., Cha J.D. (2011). Synergistic effects between silibinin and antibiotics on methicillin-resistant *Staphylococcus aureus* isolated from clinical specimens. Biotechnol. J..

[44] Shen X., Liu H., Li G., Deng X., Wang J. (2019). Silibinin attenuates *Streptococcus suis* serotype 2 virulence by targeting suilysin. J. Appl. Microbiol..

[45] Behbahani B.A., Shahidi F., Yazdi F.T., Mortazavi S.A., Mohebbi M. (2017). Antioxidant activity and antimicrobial effect of tarragon (*Artemisia dracunculus*) extract and chemical composition of its essential oil. J. Food Meas. Charact..

[46] Sahibzada M.U.K., Sadiq A., Zahoor M., Naz S., Shahid M., Qureshi N.A. (2020). Enhancement of bioavailability and hepatoprotection by silibinin through conversion to nanoparticles prepared by liquid antisolvent method. Arab. J. Chem..

[47] Yun D.G., Lee D.G. (2016). Silibinin triggers yeast apoptosis related to mitochondrial Ca^2+^ influx in *Candida albicans*. Int. J. Biochem. Cell Biol..

[48] Gowtham R., Yousuf F., Ezhilarasan D., Sambantham S., Anandan B. (2018). In vitro antifungal effects of hesperetin and silibinin. Pharmacogn. J..

[49] Umetsu T., Inoue J., Kogure T., Kakazu E., Ninomiya M., Iwata T., Takai S., Nakamura T., Sano A., Shimosegawa T. (2018). Inhibitory effect of silibinin on hepatitis B virus entry. Biochem. Biophys. Rep..

[50] Ferenci P., Scherzer T.M., Kerschner H., Rutter K., Beinhardt S., Hofer H., Schöniger–Hekele M., Holzmann H., Steindl–Munda P. (2008). Silibinin is a potent antiviral agent in patients with chronic hepatitis C not responding to pegylated interferon/ribavirin therapy. Gastroenterology.

[51] Ahmed–Belkacem A., Ahnou N., Barbotte L., Wychowski C., Pallier C., Brillet R., Pohl R.T., Pawlotsky J.M. (2010). Silibinin and related compounds are direct inhibitors of hepatitis C virus RNA-dependent RNA polymerase. Gastroenterology.

[52] Payer B., Reiberger T., Rutter K., Beinhardt S., Staettermayer A., Peck-Radosavljevic M., Ferenci P. (2010). Successful HCV eradication and inhibition of HIV replication by intravenous silibinin in an HIV–HCV coinfected patient. J. Clin. Virol..

[53] Eurich D., Bahra M., Berg T., Boas-Knoop S., Biermer M., Neuhaus R., Neuhaus P., Neumann U. (2011). Treatment of hepatitis C-virus-reinfection after liver transplant with silibinin in nonresponders to pegylated interferon-based therapy. Exp. Clin. Transplant. Off. J. Middle East Soc. Organ Transplant..

[54] Wagoner J., Morishima C., Graf T.N., Oberlies N.H., Teissier E., Pécheur E.-I., Tavis J.E., Polyak S.J. (2011). Differential in vitro effects of intravenous versus oral formulations of silibinin on the HCV life cycle and inflammation. PLoS ONE.

[55] Aghemo A., Bhoori S., Mazzaferro V., Colombo M. (2012). Failure of intravenous silibinin monotherapy to prevent hepatitis C genotype 2A liver graft reinfection. Hepat. Mon..

[56] Guedj J., Dahari H., Pohl R.T., Ferenci P., Perelson A.S. (2012). Understanding silibinin’s modes of action against HCV using viral kinetic modeling. J. Hepatol..

[57] Blaising J., Lévy P.L., Gondeau C., Phelip C., Varbanov M., Teissier E., Ruggiero F., Polyak S.J., Oberlies N.H., Ivanovic T. (2013). Silibinin inhibits hepatitis C virus entry into hepatocytes by hindering clathrin-dependent trafficking. Cell. Microbiol..

[58] Mariño Z., Crespo G., D’Amato M., Brambilla N., Giacovelli G., Rovati L., Costa J., Navasa M., Forns X. (2013). Intravenous silibinin monotherapy shows significant antiviral activity in HCV-infected patients in the peri-transplantation period. J. Hepatol..

[59] Esser-Nobis K., Romero-Brey I., Ganten T.M., Gouttenoire J., Harak C., Klein R., Schemmer P., Binder M., Schnitzler P., Moradpour D. (2013). Analysis of hepatitis C virus resistance to silibinin in vitro and in vivo points to a novel mechanism involving nonstructural protein 4B. Hepatology.

[60] Braun D., Rauch A., Durisch N., Eberhard N., Anagnostopoulos A., Ledergerber B., Metzner K., Böni J., Weber R., Fehr J. (2014). Efficacy of lead-in silibinin and subsequent triple therapy in difficult-to-treat HIV/hepatitis C virus-coinfected patients. HIV Med..

[61] Shilu Mathew S.M., Muhammad Faheem M.F., Govindaraju Archunan G.A., Muhammad Ilyas M.I., Nargis Begum N.B., Syed Jahangir S.J., Ishtiaq Qadri I.Q., Al-Qahtani M., Shiny Mathew S.M. (2014). In silico studies of medicinal compounds against hepatitis C capsid protein from north India. Bioinform. Biol. Insights.

[62] Rendina M., D’Amato M., Castellaneta A., Castellaneta N.M., Brambilla N., Giacovelli G., Rovati L., Rizzi S.F., Zappimbulso M., Bringiotti R.S. (2014). Antiviral activity and safety profile of silibinin in HCV patients with advanced fibrosis after liver transplantation: A randomized clinical trial. Transpl. Int..

[63] Braun D.L., Rauch A., Aouri M., Durisch N., Eberhard N., Anagnostopoulos A., Ledergerber B., Müllhaupt B., Metzner K.J., Decosterd L. (2015). A lead-in with silibinin prior to triple-therapy translates into favorable treatment outcomes in difficult-to-treat HIV/hepatitis C coinfected patients. PLoS ONE.

[64] Canini L., DebRoy S., Mariño Z., Conway J.M., Crespo G., Navasa M., D’Amato M., Ferenci P., Cotler S.J., Forns X. (2015). Severity of liver disease affects HCV kinetics in patients treated with intravenous silibinin monotherapy. Antivir. Ther..

[65] Dahari H., Shteingart S., Gafanovich I., Cotler S.J., D’Amato M., Pohl R.T., Weiss G., Ashkenazi Y.J., Tichler T., Goldin E. (2015). Sustained virological response with intravenous silibinin: Individualized IFN-free therapy via real-time modelling of HCV kinetics. Liver Int..

[66] Castellaneta A., Massaro A., Rendina M., D’Errico F., Carparelli S., Rizzi S.F., Thomson A.W., Di Leo A. (2016). Immunomodulating effects of the anti-viral agent Silibinin in liver transplant patients with HCV recurrence. Transplant. Res..

[67] Khan H., Paeshuyse J., Murad S., Neyts J. (2016). Assessment of the activity of directly acting antivirals and other products against different genotypes of hepatitis C virus prevalent in resource-poor countries. Antivir. Res..

[68] Malaguarnera G., Bertino G., Chisari G., Motta M., Vecchio M., Vacante M., Caraci F., Greco C., Drago F., Nunnari G. (2016). Silybin supplementation during HCV therapy with pegylated interferon-α plus ribavirin reduces depression and anxiety and increases work ability. BMC Psychiatry.

[69] Liu C.-H., Lin C.-C., Hsu W.-C., Chung C.-Y., Lin C.-C., Jassey A., Chang S.-P., Tai C.-J., Tai C.-J., Shields J. (2017). Highly bioavailable silibinin nanoparticles inhibit HCV infection. Gut.

[70] Cossiga V., Sanduzzi-Zamparelli M., Sapena V., Guarino M., Dallio M., Torrisi E., Pignata L., Federico A., Salomone F., Morisco F. (2022). Beneficial Effects of Silybin Treatment After Viral Eradication in Patients with HCV-Related Advanced Chronic Liver Disease: A Pilot Study. Front. Pharmacol..

[71] McClure J., Lovelace E.S., Elahi S., Maurice N.J., Wagoner J., Dragavon J., Mittler J.E., Kraft Z., Stamatatos L., Horton H. (2012). Silibinin inhibits HIV-1 infection by reducing cellular activation and proliferation. PLoS ONE.

[72] Romanucci V., Agarwal C., Agarwal R., Pannecouque C., Iuliano M., De Tommaso G., Caruso T., Di Fabio G., Zarrelli A. (2018). Silibinin phosphodiester glyco-conjugates: Synthesis, redox behaviour and biological investigations. Bioorg. Chem..

[73] Dai J.-P., Wu L.-Q., Li R., Zhao X.-F., Wan Q.-Y., Chen X.-X., Li W.-Z., Wang G.-F., Li K.-S. (2013). Identification of 23-(s)-2-amino-3-phenylpropanoyl-silybin as an antiviral agent for influenza A virus infection in vitro and in vivo. Antimicrob. Agents Chemother..

[74] Dutta S.K., Sengupta S., Tripathi A. (2022). In silico and in vitro evaluation of silibinin: A promising anti-*Chikungunya* agent. In Vitr. Cell. Dev. Biol.-Anim..

[75] Su Y., Wu T., Yu X.-Y., Huo W.-B., Wang S.-H., Huan C., Liu Y.-M., Liu J.-M., Cui M.-N., Li X.-H. (2022). Inhibitory effect of tanshinone IIA, resveratrol and silibinin on enterovirus 68 production through inhibiting ATM and DNA-PK pathway. Phytomedicine.

[76] Bosch-Barrera J., Martin-Castillo B., Buxó M., Brunet J., Encinar J.A., Menendez J.A. (2020). Silibinin and SARS-CoV-2: Dual targeting of host cytokine storm and virus replication machinery for clinical management of COVID-19 patients. J. Clin. Med..

[77] Sardanelli A.M., Isgrò C., Palese L.L. (2021). SARS-CoV-2 main protease active site ligands in the human metabolome. Molecules.

[78] Speciale A., Muscarà C., Molonia M.S., Cimino F., Saija A., Giofrè S.V. (2021). Silibinin as potential tool against SARS-CoV-2: In silico spike receptor-binding domain and main protease molecular docking analysis, and in vitro endothelial protective effects. Phytother. Res..

[79] Hamdy R., Mostafa A., Abo Shama N.M., Soliman S.S., Fayed B. (2022). Comparative evaluation of flavonoids reveals the superiority and promising inhibition activity of silibinin against SARS-CoV-2. Phytother. Res..

[80] Mäser P., Vogel D., Schmid C., Räz B., Kaminsky R. (2001). Identification and characterization of trypanocides by functional expression of an adenosine transporter from *Trypanosoma brucei* in yeast. J. Mol. Med..

[81] Torchelsen F.K.V.d.S., Silva T.M., Milagre M.M., Silva R.R., Reis L.E.S., Branquinho R.T., Silva G.N., de Lana M. (2021). Evaluation of the anti-*Trypanosoma cruzi* activity in vitro and in vivo of silibinin and silibinin in association to benznidazole. Parasitol. Res..

[82] Pérez-Victoria J.M., Pérez-Victoria F.J., Conseil G., Maitrejean M., Comte G., Barron D., Di Pietro A., Castanys S., Gamarro F. (2001). High-affinity binding of silybin derivatives to the nucleotide-binding domain of a *Leishmania tropica* P-glycoprotein-like transporter and chemosensitization of a multidrug-resistant parasite to daunomycin. Antimicrob. Agents Chemother..

[83] Sharma M., Sehgal R., Kaur S. (2012). Evaluation of nephroprotective and immunomodulatory activities of antioxidants in combination with cisplatin against murine visceral leishmaniasis. PLoS Neglected Trop. Dis..

[84] Olías-Molero A.I., Jiménez-Antón M.D., Biedermann D., Corral M.J., Alunda J.M. (2018). In-vitro activity of silybin and related flavonolignans against *Leishmania infantum* and *L. donovani*. Molecules.

[85] Hrčková G., Kubašková T.M., Benada O., Kofroňová O., Tumová L., Biedermann D. (2018). Differential effects of the flavonolignans silybin, silychristin and 2, 3-dehydrosilybin on *Mesocestoides vogae* larvae (cestoda) under hypoxic and aerobic in vitro conditions. Molecules.

[86] Yan Y., Li X., Zhang C., Lv L., Gao B., Li M. (2021). Research progress on antibacterial activities and mechanisms of natural alkaloids: A review. Antibiotics.

[87] Dwyer D.J., Camacho D.M., Kohanski M.A., Callura J.M., Collins J.J. (2012). Antibiotic-induced bacterial cell death exhibits physiological and biochemical hallmarks of apoptosis. Mol. Cell.

[88] Lee B., Hwang J., Lee D. (2020). Antibacterial action of lactoferricin B like peptide against *Escherichia coli*: Reactive oxygen species-induced apoptosis-like death. J. Appl. Microbiol..

[89] Erental A., Sharon I., Engelberg-Kulka H. (2012). Two programmed cell death systems in *Escherichia coli*: An apoptotic-like death is inhibited by the mazEF-mediated death pathway. PLoS Biol..

[90] Vaou N., Stavropoulou E., Voidarou C., Tsakris Z., Rozos G., Tsigalou C., Bezirtzoglou E. (2022). Interactions between medical plant-derived bioactive compounds: Focus on antimicrobial combination effects. Antibiotics.

[91] Tuli H.S., Mittal S., Aggarwal D., Parashar G., Parashar N.C., Upadhyay S.K., Barwal T.S., Jain A., Kaur G., Savla R. (2021). Path of Silibinin from diet to medicine: A dietary polyphenolic flavonoid having potential anti-cancer therapeutic significance. Semin. Cancer Biol..

[92] Wen Z., Dumas T.E., Schrieber S.J., Hawke R.L., Fried M.W., Smith P.C. (2008). Pharmacokinetics and metabolic profile of free, conjugated, and total silymarin flavonolignans in human plasma after oral administration of milk thistle extract. Drug Metab. Dispos..

[93] Hawke R.L., Schrieber S.J., Soule T.A., Wen Z., Smith P.C., Reddy K.R., Wahed A.S., Belle S.H., Afdhal N.H., Navarro V.J. (2010). Silymarin ascending multiple oral dosing phase I study in noncirrhotic patients with chronic hepatitis C. J. Clin. Pharmacol..

[94] Lo D., Wang Y.-T., Wu M.-C. (2014). Hepatoprotective effect of silymarin on di (2-ethylhexyl) phthalate (DEHP) induced injury in liver FL83B cells. Environ. Toxicol. Pharmacol..

[95] Kim Y., Kim E., Lee E., Kim J., Jang S., Kim Y., Kwon J., Kim W., Lee M. (2003). Comparative bioavailability of silibinin in healthy male volunteers. Int. J. Clin. Pharmacol. Ther..

[96] Loguercio C., Festi D. (2011). Silybin and the liver: From basic research to clinical practice. World J. Gastroenterol. WJG.

[97] Gheybi F., Khooei A., Hoseinian A., Doagooyan M., Houshangi K., Jaafari M.R., Papi A., Khoddamipour Z., Sahebkar A., Alavizadeh S.H. (2023). Alleviation of acetaminophen-induced liver failure using silibinin nanoliposomes: An in vivo study. Biochem. Biophys. Res. Commun..

[98] Durymanov M., Permyakova A., Reineke J. (2020). Pre-treatment with PLGA/silibinin nanoparticles mitigates dacarbazine-induced hepatotoxicity. Front. Bioeng. Biotechnol..

